# Remineralization of Artificial Caries-like Lesions in Permanent Enamel by MI Paste One: Comparative Physicochemical Outcomes and In Vitro Resistance to Secondary Demineralization

**DOI:** 10.3390/jfb17070333

**Published:** 2026-07-09

**Authors:** Najla F. Alsayari, Sarah S. Al-Angari, Latifa A. Alhowaish, Abdulaziz S. Alangari, Raman Bedi, Ayman M. Sulimany, Maha A. Alsarheed

**Affiliations:** 1 Department of Pediatric Dentistry and Orthodontics, College of Dentistry, King Saud University, Riyadh 11545, Saudi Arabia; lalhowaish@ksu.edu.sa (L.A.A.); asulimany@ksu.edu.sa (A.M.S.); malsarheed@ksu.edu.sa (M.A.A.); 2 Department of Restorative Dental Science, College of Dentistry, King Saud University, Riyadh 11545, Saudi Arabia; sangari@ksu.edu.sa; 3 Department of Epidemiology and Biostatistics, College of Public Health and Health Informatics, King Saud bin Abdulaziz University for Health Sciences, Riyadh 11426, Saudi Arabia; angaria@ksau-hs.edu.sa; 4 King Abdullah International Medical Research Center, Riyadh 11481, Saudi Arabia; 5 Faculty of Dentistry, Oral & Craniofacial Sciences, King’s College London, London SE1 9RT, UK; raman.bedi@kcl.ac.uk; 6 Bio-Research Chair in Dental Health, King Saud University, Riyadh 11545, Saudi Arabia

**Keywords:** casein phosphopeptide-amorphous calcium phosphate (CPP–ACP), demineralization, enamel remineralization, gloss, surface microhardness, roughness, MI Paste One (MIP-1), permanent teeth, dental caries, toothbrushing

## Abstract

Background: Patients with White spot lesions (WSLs) are seeking minimally invasive remineralizing treatment. While fluoride is the gold standard of non-invasive caries therapy, its remineralizing impact is predominantly superficial. MI Paste One (MIP-1) combines casein phosphopeptide–amorphous calcium phosphate (CPP–ACP) with 1100 ppm fluoride. This in vitro study aimed to assess the effect of MIP-1 compared with other agents. Methods: Enamel slabs were demineralized to create a caries-like lesion and randomized into five groups (*n* = 10): G1, WSL/artificial saliva (AS) (negative control); G2, deionized water brushing; G3, fluoridated toothpaste brushing; G4, MIP-1 brushing; G5, topical MI Paste application. Color change (ΔE), gloss (GU), microhardness (VHN), roughness (Ra), and surface topography (SEM) were assessed at four time points: baseline, first demineralization, treatment, and second demineralization. Results: All active agents improved microhardness after treatment. After secondary demineralization, G4 achieved the highest hardness, significantly superior to G3 (*p* = 0.016). G5 occupied a statistically intermediate position. CPP–ACP-containing agents (G4, G5) achieved color improvement that was significantly superior to controls. Conclusions: MIP-1 outperformed fluoride toothpaste in resisting secondary demineralization of permanent enamel WSLs. CPP–ACP made a measurable independent contribution to demineralization resistance in permanent enamel. These findings highlight the need for further clinical investigation of MIP-1.

## 1. Introduction

White spot lesions (WSLs) are the earliest visible sign of enamel demineralization and represent a critical therapeutic window in which lesion progression to cavitation can still be halted or reversed through non-invasive remineralization strategies. In permanent teeth, WSLs arise in two principal clinical contexts: as a consequence of active caries in adolescents and young adults with suboptimal dietary and hygiene behavior [1], and iatrogenically following fixed orthodontic appliance therapy, where systematic reviews consistently report new or enlarged WSLs in 68–73% of patients [2,3]. In both contexts, the permanent dentition carries these lesions for a lifetime, making effective non-invasive management an urgent clinical priority.

Fluoride remains the cornerstone of caries prevention and remineralization. It facilitates fluorapatite precipitation at the enamel surface and within the lesion body, reducing solubility and promoting crystallographic repair [4,5]. However, its remineralization potential depends on the availability of salivary calcium and phosphate ions, which fluoride does not supply. This creates a ceiling effect, particularly in the deeper subsurface zones of established WSLs [6].

Casein phosphopeptide–amorphous calcium phosphate (CPP–ACP)—the active ingredient in MI Paste (GC America)—was developed to address the ceiling effect limitation of fluoride. By stabilizing supersaturated concentrations of calcium and phosphate at the enamel surface, CPP–ACP creates a sustained ionic reservoir that drives mineral diffusion into enamel microporosities [7,8]. When co-administered with fluoride, a synergistic effect has been demonstrated, with the CPP–ACP reservoir enabling deeper fluorapatite deposition than achieved by fluoride alone [9]. MI Paste One (GC America Inc., Alsip, IL, USA) combines these two mechanisms in a single daily-use toothpaste formulation [10].

The available literature regarding the clinical and laboratory efficacy of combined CPP–ACP and fluoride therapies presents conflicting observations. Recent reviews and experimental studies show that remineralizing agents can produce beneficial changes in WSLs, but their relative effects vary according to substrate, lesion model, application protocol, and outcome measure [11,12,13,14]. Clinical reviews generally support CPP–ACP and casein phosphopeptide-amorphous calcium phosphate fluoride (CPP–ACPF) as potentially useful adjuncts for WSL management, although results are inconsistent and do not always demonstrate superiority over fluoride-only approaches [11,12]. In vitro micro-CT and SEM studies also indicate that fluoride- and calcium-phosphate-based products may differ in surface versus subsurface effects [13,14]. These conflicting observations highlight the need to evaluate not only immediate rehardening but also the durability of remineralized enamel under a subsequent acid challenge.

Despite commercial availability, the direct efficacy of MI Paste One on human permanent enamel remains unexplored. One previous laboratory investigation utilizing a dynamic pH-cycling model on bovine enamel reported that MI Paste One did not demonstrate superior remineralizing efficacy compared with a conventional fluoride toothpaste [15]. However, bovine enamel differs substantially from human permanent enamel in structural porosity and mineral diffusion dynamics. Furthermore, while pH-cycling evaluates continuous ionic flux, the present study utilizes a static model designed to isolate cumulative mineral uptake prior to an absolute secondary acid challenge. Evaluating these distinct variables provides a highly novel understanding of whether CPP–ACP makes a meaningful independent contribution to remineralization in permanent teeth, and whether its combination with fluoride outperforms either agent alone.

Therefore, this study aimed to evaluate the in vitro effects of MI Paste One, fluoridated toothpaste, and topical MI Paste on artificial caries-like lesions in permanent premolar enamel, and to assess the capacity of treated lesions to resist subsequent acid challenge. Surface microhardness was designated as the primary quantitative endpoint to evaluate structural mineral recovery, while color, gloss, and surface roughness served as secondary aesthetic and topographical outcomes. The null hypotheses were that the experimental treatments would yield no significant differences in (i) the primary and secondary endpoints following treatment, and (ii) the subsequent resistance of the lesions to secondary demineralization.

## 2. Materials and Methods

### 2.1. Ethical Approval and Study Design

This in vitro study was approved by the Institutional Review Board of King Saud University (registration #E-23-8152) and the College of Dentistry Research Center (registration #PR-0154) and was conducted in accordance with the Declaration of Helsinki.

**Figure 1:**
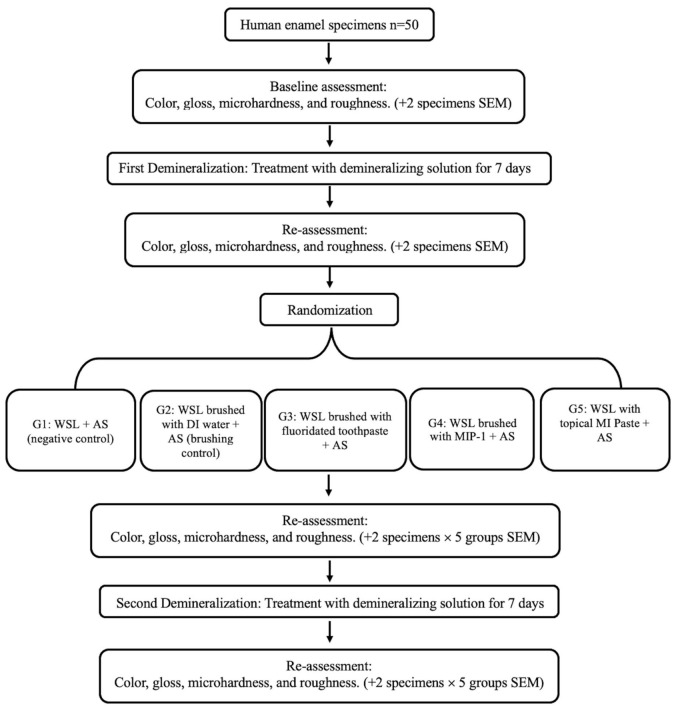
Study design and SEM specimen allocation.

Enamel slabs (3 × 3 × 2 mm) were prepared from the buccal and lingual surfaces of sound human permanent premolar teeth (informed consent obtained from tooth donors). A total of 74 specimens were prepared: 50 for quantitative outcome measurement (*n* = 10 per group, maintained without attrition throughout all four stages) and 24 additional specimens allocated exclusively to scanning electron microscopy (SEM) imaging. All specimens were stored in 0.1% thymol at 4 °C until use. Specimens were sectioned using a low-speed diamond saw (Isomet, Buehler, Lake Bluff, IL, USA), embedded in epoxy resin, surface-flattened using sequential 500–4000 grit silicon carbide paper (MD Fuga, Struers Inc., Cleveland, OH, USA), polished to 1 µm with diamond suspension, and cleaned with Micro-90 (International Products Corporation, Burlington, NJ, USA) [16]. The polishing protocol removes the aprismatic surface layer, exposing the highly mineralized prismatic enamel core; this accounts for the higher-than-typical baseline Vickers microhardness values observed (discussed below). A stratified allocation approach was employed. The specimens were assigned to one of the five experimental groups (*n* = 10) based on their baseline microhardness values. This ensured an equal and homogeneous distribution of initial enamel hardness across all groups prior to the demineralization and treatment phases [17] (Figure 1).

Due to the physical differences in the application protocols, the outcome assessor was not blinded to the group assignments. However, potential operator bias was minimized by relying strictly on objective, instrument-derived quantitative measurements for all primary and secondary outcomes.

### 2.2. Generation of Artificial Caries-like Lesions

Caries-like lesions were created by immersing specimens in a demineralizing solution for 7 days at 37 °C. The solution contained 0.1 mM lactic acid, 4.1 mM CaCl_2_, and 8.0 mM KH_2_PO_4_, adjusted to pH 5.0 using Sodium hydroxide as a buffering agent [16]. The solution was not stirred or replaced during this period to maintain static diffusion conditions.

### 2.3. Artificial Saliva and Treatment Groups

Artificial saliva (AS) consisted of 1.45 mM CaCl_2_·2H_2_O, 5.40 mM KH_2_PO_4_, 28.4 mM NaCl, and 14.9 mM KCl at pH 7.0, replaced daily [18]. This AS composition provides a mildly supersaturated solution with respect to hydroxyapatite; as all groups were stored under identical AS conditions, any background remineralizing effect is expected to be uniform across groups. Specimens were randomized into five groups (*n* = 10) as described in Table 1.

### 2.4. Simulated Toothbrushing Protocol

Toothpaste slurries were prepared by mixing 20 g of toothpaste with 20 mL of AS [19]. Specimens in G2, G3, and G4 were brushed in an automated brushing machine (ZM-3.8, SD Mechatronik, Feldkirchen-Westerham, Germany) using soft toothbrushes (Yara Special Soft) under 200 g load for 230 strokes per cycle, twice daily for 7 days [20,21]. This brushing protocol is equivalent to approximately 11 months of clinical brushing of a specific tooth surface [22]. During the 7-day experimental period, specimens in the brushing groups were brushed with their assigned slurries twice daily for 2 min per session, resulting in a total of 28 min (1680 s) of direct brushing contact per specimen surface. While the American Dental Association recommends a total brushing duration of 2 min twice daily for the entire dentition [23], the established literature documents that a single tooth surface receives only about 5 s of direct brush contact per day during a standard clinical routine [24]. By concentrating 1680 s of brushing directly onto the isolated enamel specimens in vitro, this 7-day protocol effectively simulates approximately 336 days (roughly 11 months) of clinical brushing wear on a single tooth surface.

G5 received once-daily topical MI Paste application per manufacturer’s instructions (finger/tray application for 3 min, without mechanical brushing), replicating the recommended clinical protocol for this product. This intentional design difference—brushing for G3/G4 versus topical application for G5—reflects the different delivery mechanisms of the two product types and is acknowledged as a between-group confounding factor limiting direct chemical comparison. After each cycle, all specimens were rinsed with deionized water, air-dried, and stored in fresh AS at 37 °C. Outcome measurements were performed on air-dried specimens after removal from storage after the 7-day treatment period.

### 2.5. Outcome Measurements

#### 2.5.1. Color (ΔE)

Surface color was assessed using a spectrophotometer (LabScan XE, Hunter Associates Laboratory Inc., Reston, VA, USA) under the CIE L*a*b* system at four time points: baseline, post-demineralization, post-remineralization, and post-secondary-demineralization. Total color difference (ΔE) was calculated as follows:

ΔE = [(ΔL)^2^ + (Δa)^2^ + (Δb)^2^]^1/2^

The specific color changes were determined at each stage as follows [16]:

At first, demineralization:

ΔE_0_ = ΔE_Demin_ − ΔE_Baseline_

Post-treatment:

ΔE_1_ = ΔE_treatment_ − ΔE_Demin_

Post-secondary-demineralization:

ΔE_2_ = ΔE_Second demine_ − ΔE_treatment_

The values are expressed relative to the preceding stage [16]. A color change of ΔE ≥ 3.3 was considered clinically perceptible [25]. Individual L* (lightness), a* (red–green), and b* (blue–yellow) component data are provided in Appendix A.

#### 2.5.2. Gloss (GU)

Surface gloss was measured using a glossmeter (Novo-Curve, Rhopoint Instruments, East Sussex, UK) at a 60° projection angle, as per ISO 2813 [26]. Three measurements were taken per specimen per stage; the mean GU was recorded. The instrument was calibrated against a polished black glass reference standard (100 GU); highly polished mineral surfaces can yield values exceeding 100 GU at 60° under this calibration.

#### 2.5.3. Vickers Microhardness (VHN)

Vickers microhardness was assessed using an Innovatest system (Innovatest Europe BV, Maastricht, The Netherlands) at a 50 g load for 11 s [27]. Three indentations were made per specimen per stage, placed ≥100 µm apart; the mean VHN was recorded.

#### 2.5.4. Surface Roughness (Ra)

Surface roughness was assessed using a non-contact optical profilometer (Contour GT-K, Bruker Inc., Billerica, MA, USA). The mean of three Ra readings (µm) per specimen per stage was recorded [28].

#### 2.5.5. Scanning Electron Microscopy (SEM)

Surface morphology was qualitatively evaluated using SEM (JSM-6360LV, JEOL USA, Inc., Peabody, MA, USA) at 1500× magnification. A total of 24 dedicated enamel specimens were gold-sputter-coated and imaged. These specimens were processed simultaneously with the primary samples used for quantitative microhardness testing, undergoing the exact same standardized protocols. To capture morphological changes across the study timeline, the 24 specimens were systematically allocated as follows: two specimens were imaged at baseline (prior to group allocation), and two specimens were imaged following the initial demineralization phase (representing uniform demineralization before treatment assignment). The remaining 20 specimens were then distributed equally between the five experimental groups (*n* = 4 per group). Within each experimental group, two specimens were evaluated immediately post-treatment, and the final two specimens were evaluated following the secondary demineralization challenge [29].

### 2.6. Statistical Analysis

Sample size was calculated based on the primary outcome variable (Vickers microhardness) using G*Power v3.1.9.7. Assuming an expected effect size of 0.25, a power of 90%, and an alpha level of 0.05, the analysis yielded a minimum of 10 specimens per group. Statistical analyses were conducted using SAS v9.4 (SAS Institute Inc., Cary, NC, USA). Prior to inferential testing, normality was assessed using the Shapiro-Wilk test and homogeneity of variance was evaluated using Levene’s test. Data were approximately normally distributed in the majority of subgroups; isolated minor departures were non-systematic, and ANOVA is robust to such violations under balanced designs (*n* = 10 per group). One-way ANOVA was used to compare differences among groups at each time point (Group as between-subjects factor); pairwise comparisons used Tukey’s HSD test. Within-group changes over time were assessed using repeated-measures ANOVA and Tukey–Kramer adjusted pairwise comparisons. Sphericity was formally assessed using Mauchly’s test; sphericity was violated for all four outcome measures (all *p* ≤ 0.0003), and the Greenhouse–Geisser correction was applied to all within-subjects tests. All analyses were conducted in SAS v9.4 (SAS Institute Inc., Cary, NC, USA) with α = 0.05.

## 3. Results

### 3.1. Color

Initial demineralization produced a significant and uniform increase in color values across all groups (ΔE range: 9.4–11.1; *p* < 0.05), all exceeding the 3.3 threshold for clinical perceptibility, with no intergroup differences. Following treatment, G4 and G5 achieved significantly greater color improvement than controls (*p* ≤ 0.040), whereas G3 was statistically indistinguishable from the treatment groups and the controls. After secondary demineralization, there were no intergroup differences. Full color data are presented in Table 2.

### 3.2. Gloss

Gloss values were lower after demineralization and recovered following treatment in G3–G5. No differences between or within groups reached statistical significance at any stage (Table 3). Baseline values (164–169 GU) exceed the nominal 100 GU calibration reference; this is discussed below.

### 3.3. Surface Microhardness

Baseline microhardness was comparable across all groups (approximately 470 VHN). Demineralization significantly reduced hardness in all groups (*p* < 0.0001), with no intergroup differences, confirming uniform lesion formation. Following treatment, G3–G5 all showed significant rehardening compared with post-demineralization and control groups (*p* < 0.0001), with no significant differences between the three treatment groups at this stage (G3 = G4 = G5).

After secondary demineralization, G4 retained the highest microhardness (293 ± 62 VHN), significantly superior to G3 (218 ± 38 VHN; *p* = 0.016) and both control groups. G5 occupied a statistically intermediate position (247 ± 38 VHN), not significantly different from either G4 or G3 (Table 4). Analysis of VHN loss from post-treatment to post-secondary demineralization (Table 5) showed that among the treatment groups, G3 sustained the greatest absolute and proportional loss (105 VHN [32%]), while G4 demonstrated the lowest (79 VHN [21%]), indicating a trend toward enhanced mineral stability after acid challenge. Control groups G1 and G2 demonstrated smaller absolute losses (39 VHN [20%] and 37 VHN [19%], respectively), consistent with their substantially lower post-treatment starting values.

### 3.4. Surface Roughness

**Figure 2:**
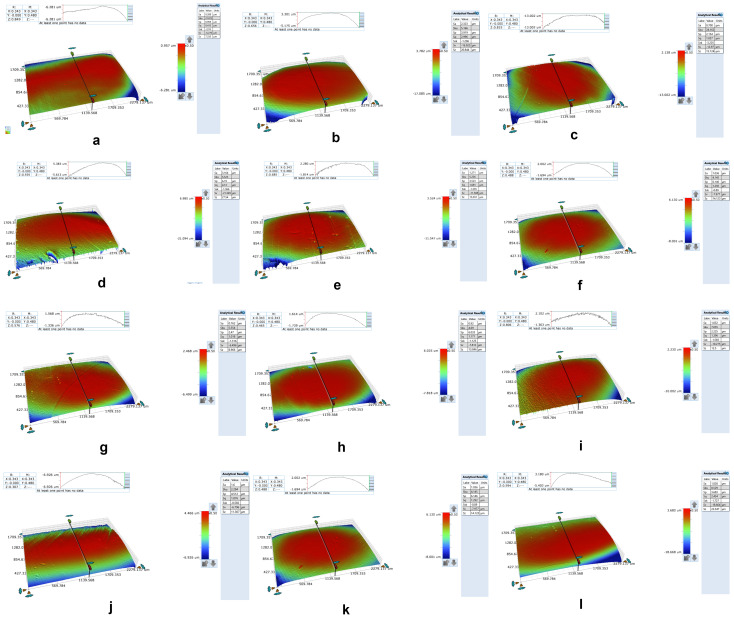
Profilometer images. (**a**): At baseline, the Ra is 0.28 (**b**): At the first demineralization, the Ra value increased to 2.3, indicating increased surface roughness. (**c**) The post-treatment Ra values for the white spot lesions (WSLs) without treatment (G1) were significantly lower than the first demineralization (0.7). For G2 (WSLs brushed with deionized (DI) water), (**d**), G3 (WSLs brushed with fluoridated toothpaste), (**e**), G4 (WSLs brushed with MI Paste One (MIP-1)), (**f**), and G5 (WSLs with topical MI Paste; (**g**) the Ra value remained high, with no statistically significant difference from the first demineralization. Post-secondary demineralization showed no statistically significant difference from the post-treatment values for G1 (**h**), G2 (**i**), G3 (**j**), G4 (**k**), and G5 (**l**). The decrease in Ra value for G2 was almost significant.

Surface roughness increased significantly after demineralization in all groups (*p* < 0.05). Following treatment, only G1 showed a significant reduction versus post-demineralization (*p* < 0.05); no intergroup differences were significant. After secondary demineralization, only G2 showed a borderline non-significant roughness reduction (*p* = 0.0505). No statistically significant intergroup differences were observed at any stage (Table 6; Figure 2).

### 3.5. Scanning Electron Microscopy

**Figure 3:**
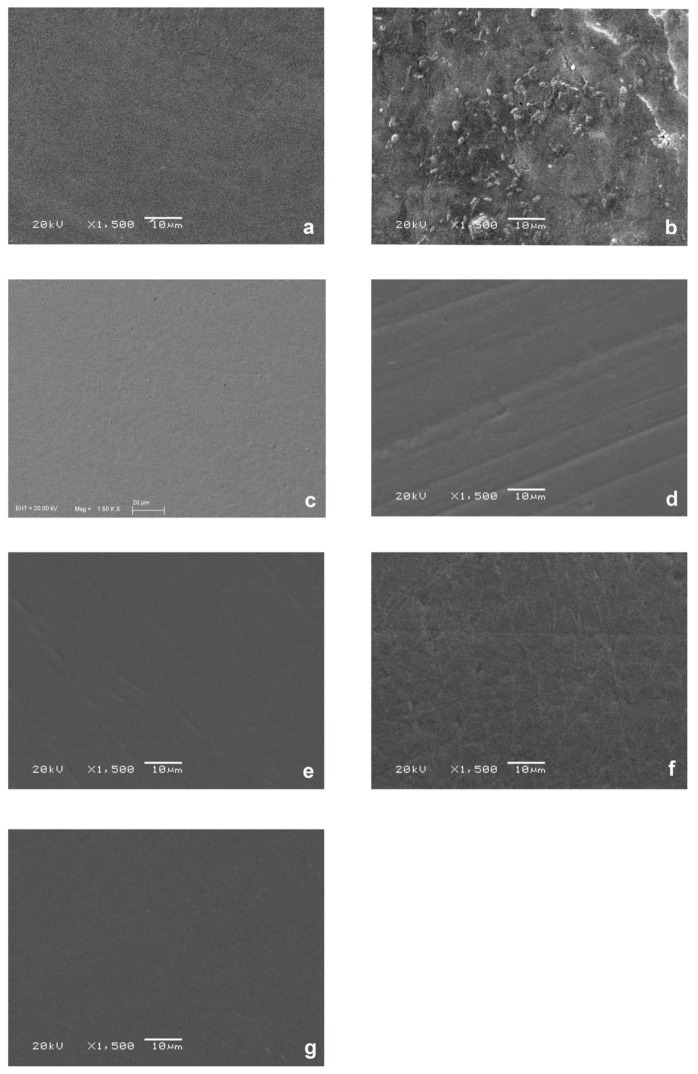
Scanning electron microscope (SEM) images at 1500× magnification. (**a**): At baseline, showing smooth, intact enamel surfaces, (**b**): At the first demineralization, exposed enamel pores and pitted surface. Post-treatment G1 (white spot lesions (WSLs); (**c**): G2 (WSLs brushed with deionized (DI) water; (**d**), G3 (WSLs brushed with fluoridated toothpaste; (**e**), G4 (WSLs brushed with MI Paste One (MIP-1); (**f**), and G5 (WSLs subjected to MI Paste; (**g**) samples, showing fine irregularities, brushing strikes and an irregular surface, an irregular but intact surface, minor brushing artifacts with an intact surface, and intact surface with deposits consistent with residual CPP–ACP, respectively.

**Figure 4:**
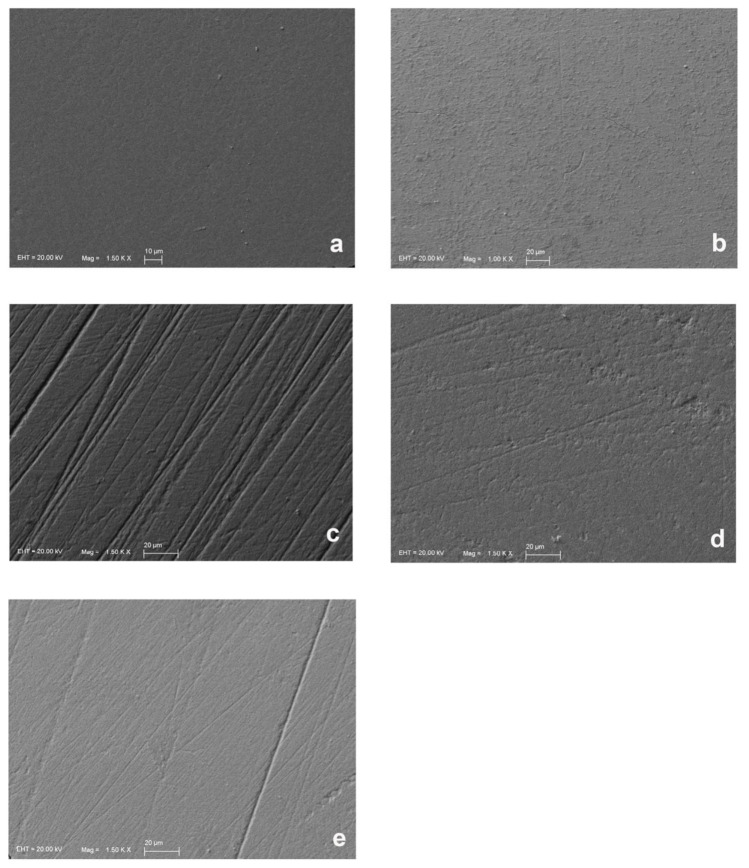
Scanning electron microscope (SEM) images at 1500× magnification post-secondary demineralization. (**a**): G1 (white spot lesions (WSLs)) demonstrating irregular, cracked surface. (**b**): G2 (WSLs brushed with deionized (DI) water) showing irregular, cracked surface. (**c**): G3 (WSLs brushed with fluoridated toothpaste) showing irregular but intact enamel. (**d**): G4 (WSLs brushed with MI Paste One (MIP-1)) demonstrating surface deposits and minimal microporosity, and (**e**): G5 (WSLs subjected to MI Paste) showing some surface cracking and surface deposits.

Baseline SEM images confirmed smooth, intact enamel surfaces with regular prismatic architecture and visible prism boundaries in all groups. Post-demineralization images revealed enamel porosity and characteristic keyhole-shaped prism dissolution patterns. Following treatment, G1 showed persistent surface irregularities, and G2 showed prominent brushing striations. G3 and G4 showed largely intact surfaces with minor brushing artifacts, with G4 appearing visually smoother than G3. G5 showed intact enamel with surface deposits. Post-secondary demineralization, G1 and G2 showed irregular, cracked surfaces. G3 showed irregular but intact enamel; G4 retained surface deposits and minimal microporosity, while G5 showed some surface cracking alongside residual deposits. (Figure 3 and Figure 4).

## 4. Discussion

The findings of this study have implications for clinicians and patients who seek a minimally invasive remineralization option that improves the optical and physical properties of enamel. This study provides the first direct comparative in vitro evaluation of MI Paste One (MIP-1) compared with other remineralizing agents—fluoride toothpaste alone and fluoride-free CPP–ACP (MI Paste)—using human permanent premolar enamel as the experimental substrate. Three findings are of particular scientific and clinical significance.

The primary finding is the superior hardness retention of G4 after secondary demineralization (293 VHN), which was significantly higher than G3 (218 VHN; *p* = 0.016). Crucially, the VHN loss analysis (Table 5) reveals that G3 sustained the greatest absolute and proportional hardness loss on secondary acid challenge (105 VHN; 32%), substantially exceeding G4 (79 VHN; 21%). However, it is important to note that these calculated loss metrics are reported as descriptive observational trends to illustrate the relative magnitude of demineralization, rather than as inferentially tested statistical differences. This is mechanistically important: fluoride-mediated remineralization preferentially deposits fluorapatite at the enamel surface and within the outer lesion zone, forming a dense but potentially thin mineral layer that, while hard at post-treatment measurement, may be susceptible to rapid dissolution on renewed acid exposure if the subsurface mineral gradient is not adequately restored [6]. CPP–ACP co-administration, by sustaining a deeper calcium and phosphate reservoir, promotes more distributed subsurface mineralization that may be inherently more stable under acid challenge [9,30]. This interpretation is qualitatively supported by the SEM finding of persistent surface deposits and exhibited minimal microporosity in G4 after secondary demineralization. Future micro-CT studies should compare subsurface mineral density gradients between G3 and G4 after sequential acid challenge.

CPP–ACP alone (G5) demonstrated a statistically intermediate position in permanent enamel after secondary demineralization (247 VHN), sharing statistical equivalence with G4 and G3. This upward shift in permanent enamel suggests CPP–ACP alone confers a greater independent contribution to demineralization resistance in the permanent dentition, likely reflecting the lower organic fraction and more regular crystallographic structure of permanent enamel, facilitating deeper CPP–ACP-mediated mineral deposition [30,31,32]. These results more accurately suggest that CPP-ACP alone merits further clinical investigation as a promising, fluoride-free remineralizing option.

Furthermore, the role of the artificial saliva model must be acknowledged as a critical background factor. Because the artificial saliva medium is supersaturated with calcium and phosphate ions, it acts as an active remineralizing agent across all experimental groups. Within G1, AS significantly increased surface hardness compared with the second demineralization phase. This suggests that AS offers a mild remineralizing effect [33] and the lowest inhibitory protection against subsequent acid challenges [34]. Additionally, the pronounced remineralization observed in the active treatment groups (G3–G5) should not be viewed as the isolated efficacy of the applied agents alone, but rather as a synergistic interaction between the topical treatments and the continuous ionic supply provided by the artificial saliva storage medium.

In permanent enamel, fluoride toothpaste alone (G3) did not achieve color improvement statistically separable from the untreated control, whereas MI Paste One (G4) and MI Paste (G5) did. However, G3 exceeded the 3.3 threshold for clinical perceptibility (5.3 ± 1.9) compared with controls (2.9 ± 0.9 and 2.8 ± 1.7). CPP–ACP’s sustained subsurface calcium and phosphate delivery appears necessary to achieve detectable optical recovery in permanent enamel, where the lesion body is deeper and organic content is lower [35,36]. An alternative mechanism must also be acknowledged: CPP–ACP particles from MI Paste formulations may physically occupy surface microporosities, altering light scattering independently of true mineral crystal deposition. Individual L*, a*, and b* data (Appendix A) confirm that ΔE was driven predominantly by the L* axis in all groups, reflecting changes in surface opacity consistent with WSL formation and partial remineralization. Importantly, while these optical modifications offer a pronounced immediate optical improvement, this aesthetic masking effect was temporary, as intergroup color differences did not persist following the secondary demineralization challenge. Consequently, these visual improvements should be interpreted as supportive findings rather than evidence of lasting aesthetic superiority against subsequent acid exposure. Micro-CT analysis would allow deconvolution of these effects in future work.

A previous study reported that MI Paste One produced the lowest percentage surface microhardness recovery in a bovine enamel pH-cycling model (mean 4.3%), compared with conventional fluoride toothpaste (26.2%) [15]. Several methodological differences likely account for this discrepancy. First, it is important to highlight that variations between study findings are inherently dependent on the specific experimental substrates utilized; bovine and human permanent enamel differ in prism architecture, organic content, and surface chemistry [31,32], yet both present distinct methodological advantages in cariology research. Second, the pH-cycling model of Lippert and Gill [15] evaluates agents under repeated acid challenge concurrent with application. In contrast, the static model utilized in the present study isolates the cumulative remineralizing phase prior to a single secondary acid challenge. Therefore, the differing remineralization dynamics observed reflect the unique characteristics and kinetic environments of the chosen models, rather than indicating the methodological superiority of one protocol over another. Third, percentage surface microhardness recovery primarily captures initial mineral uptake, whereas the present study emphasizes absolute resistance to secondary demineralization, metrics that evaluate distinct phases of the caries process.

Gloss was not significantly affected by any treatment, which is consistent with the published in vitro literature [37]. Mechanistically, CPP–ACP and fluoride-mediated mineral deposition occur at the nanometer-to-micrometer scale, below the threshold detectable by specular reflectance at 60° incidence; gloss is therefore not an appropriate primary endpoint for subsurface remineralization studies. Surface roughness similarly showed no significant intergroup differences; roughness elevation persisting in the brushed groups likely reflects micro-abrasion tracks on partially softened enamel, a recognized phenomenon with mechanical brushing protocols on demineralized substrates [20,21]. Consequently, given the absence of statistically significant variation, surface gloss and roughness parameters were considered as ancillary outcomes, serving primarily as supportive findings rather than primary indicators of remineralization efficacy.

The baseline gloss values reported (164–169 GU at 60°) exceed the nominal 100 GU glass calibration reference. Surfaces more reflective than the reference glass—physically possible for diamond-polished mineralized specimens—can yield values exceeding 100 GU under ISO 2813. The baseline Vickers microhardness values (~470 VHN) reflect the standardized polishing protocol (sequential diamond suspension to 1 µm), which removes the aprismatic surface layer and exposes the harder prismatic enamel core; comparable values have been reported for similarly prepared permanent enamel specimens [16,17]. Both measurements warrant independent cross-calibration in future work.

The cost-effectiveness of these remineralizing therapies must be considered alongside their chemical efficacy. From a clinical translation perspective, conventional fluoride dentifrice remains the most universally accessible and highly cost-effective baseline treatment for caries management and white spot lesion intervention. Conversely, CPP–ACP-based therapies represent a higher initial financial investment for the patient. However, the formulation of MI Paste One—which integrates both the cleaning agents, fluoride, and CPP–ACP into a single daily-use dentifrice—may offer a superior clinical cost-benefit ratio compared with the traditional regimen of purchasing a standard toothpaste and a supplementary topical CPP–ACP cream.

This study has the inherent limitations of in vitro methodology that cannot entirely replicate the dynamic complexities of the human oral cavity: the absence of a salivary pellicle, biofilm dynamics, thermal cycling, and variable dietary acid exposure limit extrapolation to the clinical context [38]. The intentional design difference between G5 (topical, no brushing) and G3/G4 (brushed) represents a delivery-mode confound that precludes attribution of G5 effects purely to CPP–ACP chemistry, which was necessary to simulate the manufacturer’s clinical instructions for each product. The static remineralization model does not simulate the repeated acid challenge of a cariogenic diet; pH-cycling designs would better model competitive demineralization–remineralization dynamics [15]. Consequently, these in vitro findings serve as foundational evidence that warrants further validation through long-term in situ or in vivo clinical trials. Future work should include micro-CT analysis to quantify subsurface mineral density, backscattered electron SEM for more informative surface characterization, longer acid cycling regimens, and randomized clinical trials in caries-active adult or post-orthodontic patients using quantitative light-induced fluorescence (QLF).

## 5. Conclusions

Under the tested in vitro conditions, MI Paste One (G4) exhibited the most favorable post-challenge microhardness values, suggesting enhanced resistance to secondary demineralization of permanent enamel white spot lesions compared with standard fluoride toothpaste (G3), supporting the hypothesis that the synergistic effect of CPP–ACP and fluoride may provide better resistance to subsequent acid challenges. However, MI Paste One (G4) did not show a statistically significant difference from the topical MI Paste (G5), which remained statistically intermediate between G3 and G4. All treatments resulted in significant color recovery and surface rehardening compared with the control conditions. Gloss and surface roughness were not significantly affected by any treatment, which is consistent with the insensitivity of these endpoints to subsurface mineral changes. While these in vitro findings support further clinical investigation of MI Paste One for permanent dentition white spot lesion management, randomized clinical trials are required before any definitive clinical practice guidelines can be proposed.

## Figures and Tables

**Table 1:** Treatment groups.

Group	Label	Treatment Protocol	Active Agent(s)
G1	Negative control	WSL + AS (no treatment)	None
G2	Brushing control	WSL brushed with deionized water + AS	None
G3	Fluoridated toothpaste	WSL brushed with fluoridated toothpaste + AS	1100 ppm F
G4	MI Paste One	WSL brushed with MI Paste One + AS	1100 ppm F + CPP–ACP
G5	MI Paste (topical)	WSL with topical MI Paste (no brushing) + AS	CPP–ACP (fluoride-free)

WSL: white spot lesion; AS: artificial saliva; CPP–ACP: casein phosphopeptide–amorphous calcium phosphate. Fluoridated toothpaste: Crest Complete Anticavity Fluoride Toothpaste (Procter & Gamble, Cincinnati, OH, USA; active ingredient: sodium fluoride 0.243% [1100 ppm F]). MI Paste One: GC America Inc., Alsip, IL, USA; active ingredient CPP–ACP 10% *w*/*w* [Recaldent] and 1100 ppm F. MI Paste: GC America Inc, Alsip, IL, USA; active ingredient CPP–ACP 10% *w*/*w* [Recaldent]. G2 is designated a brushing control rather than a positive control as it contains no active remineralizing agent and serves to isolate the mechanical effect of brushing from the chemical effects of G3–G5.

**Table 2:** Color change (ΔE; mean ± SD) across the three transitional study stages.

Groups	ΔE_0_	ΔE_1_	ΔE_2_
G1	10.3 ± 4.2 a	2.9 ± 0.9 a	5.8 ± 1.9 a
G2	9.4 ± 4.4 a	2.8 ± 1.7 a	7.4 ± 1.6 a
G3	10.6 ± 2.6 a	5.3 ± 1.9 b,a	4.9 ± 1.8 a
G4	11.1 ± 4.1 a	7.0 ± 3.0 b	5.6 ± 2.9 a
G5	10.8 ± 3.6 a	6.2 ± 1.5 b	7.0 ± 1.9 a

Different lowercase letters represent statistically significant intergroup differences within a stage (column; *p* < 0.05). ΔE_0_: first demineralization—baseline, ΔE_1_: treatment—first demineralization, ΔE_2_: second demineralization—treatment. ΔE is a positive scalar (Euclidean distance in color space); directionality is captured by ΔL* (negative = darkening/remineralization) in Appendix A Table A1.

**Table 3:** Surface gloss (GU; mean ± SD) at the four study stages.

Groups	Baseline	First Demineralization	Post-Treatment	Second Demineralization
G1	164 ± 13	160 ± 13	158 ± 14	148 ± 16
G2	167 ± 14	158 ± 15	159 ± 18	152 ± 17
G3	169 ± 18	157 ± 16	170 ± 16	162 ± 18
G4	164 ± 18	156 ± 17	166 ± 16	160 ± 15
G5	169 ± 18	161 ± 16	169 ± 16	161 ± 12

No statistically significant differences were observed between or within groups at any stage (*p* > 0.05). See Discussion for interpretation of values > 100 GU.

**Table 4:** Surface microhardness (VHN; mean ± SD) at the four study stages.

Groups	Baseline	First Demineralization	Post-Treatment	Second Demineralization
G1	471 ± 3 A,a	190 ± 38 B,C,a	201 ± 40 B,a	162 ± 31 C,a
G2	473 ± 7 A,a	190 ± 30 B,a	196 ± 27 B,a	160 ± 29 C,a
G3	472 ± 3 A,a	185 ± 30 B,a	323 ± 30 C,b	218 ± 38 D,b
G4	473 ± 3 A,a	188 ± 28 B,a	372 ± 54 C,b	293 ± 62 D,c
G5	472 ± 2 A,a	188 ± 34 B,a	342 ± 45 C,b	247 ± 37 D,b,c

Different uppercase letters represent statistically significant within-group differences across stages (row; *p* < 0.05), assessed by repeated-measures ANOVA. Different lowercase letters represent statistically significant intergroup differences within a stage (column; *p* < 0.05), assessed by one-way ANOVA. G4 uniquely holds letter c at the second demineralization; G5 holds b,c (an intermediate position).

**Table 5:** VHN loss from post-treatment to the post-secondary demineralization stage.

Groups	Post-Treatment VHN	Post-Secondary-Demineralization VHN	VHN Loss (Absolute)	VHN Loss (%)
G1	201	162	39	20
G2	196	160	37	19
G3	323	218	105 *	32 *
G4	372	293	79	21
G5	342	247	96	28

* G3 demonstrates the greatest absolute and proportional VHN loss, indicating that fluorapatite deposited by fluoride alone is more susceptible to secondary acid challenge than mineral deposited with CPP–ACP co-administration (G4). Note: Percentage values for VHN loss are descriptive representations of data trends; primary statistical conclusions are based on the raw VHN data analysis presented in Table 4.

**Table 6:** Surface roughness (Ra, µm; mean ± SD) at the four study stages.

Groups	Baseline	First Demineralization	Post-Treatment	Second Demineralization
G1	0.5 ± 0.2 A,a	1.0 ± 0.9 B,a	0.6 ± 0.3 A,a	0.6 ± 0.3 A,a
G2	0.5 ± 0.1 A,a	1.3 ± 1.2 B,a	1.4 ± 1.2 B,a	0.8 ± 0.6 B,a
G3	0.5 ± 0.2 A,a	1.3 ± 0.5 B,a	1.3 ± 0.3 B,a	1.3 ± 0.6 B,a
G4	0.4 ± 0.1 A,a	1.0 ± 0.5 B,a	1.0 ± 0.4 B,a	1.0 ± 0.4 B,a
G5	0.5 ± 0.2 A,a	1.2 ± 0.9 B,a	1.0 ± 0.9 B,a	1.2 ± 0.9 B,a

Different uppercase letters indicate significant within-group differences across stages (row; *p* < 0.05), assessed by repeated-measures ANOVA. Different lowercase letters indicate significant differences between groups within a stage (column; *p* < 0.05), assessed by one-way ANOVA.

## Data Availability

The data supporting the findings of this study are available from the corresponding author (N.F.A.; najla.alsayari@outlook.com) upon reasonable request.

## Abbreviations

The following abbreviations are used in this manuscript:

AS	Artificial saliva
WSL	White spot lesion
CPP–ACP	Casein phosphopeptide–amorphous calcium phosphate
SEM	Scanning electron microscopy
KH_2_PO_4_	Potassium dihydrogen phosphate
CIE	Commission Internationale de l’Eclairage
ANOVA	Analysis of variance
DI	Deionized water

## Appendix A

**Table A1:** L* (lightness) value across stages.

Groups	L*_0_	L*_1_	L*_2_
G1	8.2	−2.4	8.2
G2	6.6	−1.7	6.8
G3	6.8	−4.5	4.7
G4	7.7	−5.6	5.0
G5	7.4	−4.9	6.3

L*_0_: first demineralization—baseline, L*_1_: treatment—first demineralization, L*_2_: second demineralization—treatment.

**Table A2:** a* value across stages.

Groups	a*_0_	a*_1_	a*_2_
G1	0.2	−0.3	0.2
G2	−0.5	0.2	−0.3
G3	−0.7	0.5	0.0
G4	−0.6	0.4	−0.4
G5	−0.6	0.3	−0.4

a*_0_: first demineralization—baseline, a*_1_: treatment—first demineralization, a*_2_: second demineralization—treatment.

**Table A3:** b* value across stages.

Groups	b*_0_	b*_1_	b*_2_
G1	−5.6	0.2	−5.6
G2	−5.4	1.9	−1.7
G3	−7.3	2.1	−1.3
G4	−7.4	3.0	−2.1
G5	−7.1	2.6	−2.9

b*_0_: first demineralization—baseline, b*_1_: treatment—first demineralization, b*_2_: second demineralization—treatment.
